# Evidence for a mitochondrial localization of the retinoblastoma protein

**DOI:** 10.1186/1471-2121-10-50

**Published:** 2009-06-25

**Authors:** Ioana Ferecatu, Nathalie Le Floch, Marie Bergeaud, Aida Rodríguez-Enfedaque, Vincent Rincheval, Lisa Oliver, François M Vallette, Bernard Mignotte, Jean-Luc Vayssière

**Affiliations:** 1 Laboratoire de génétique et biologie cellulaire – CNRS UMR 8159, Université de Versailles Saint-Quentin-en-Yvelines, Versailles, France; 2 Laboratoire de génétique moléculaire et physiologique, Ecole Pratique des Hautes Etudes, Versailles, France; 3 INSERM U601, Faculté de Médecine – Université de Nantes, Nantes, France

## Abstract

**Background:**

The retinoblastoma protein (Rb) plays a central role in the regulation of cell cycle, differentiation and apoptosis. In cancer cells, ablation of Rb function or its pathway is a consequence of genetic inactivation, viral oncoprotein binding or deregulated hyperphosphorylation. Some recent data suggest that Rb relocation could also account for the regulation of its tumor suppressor activity, as is the case for other tumor suppressor proteins, such as p53.

**Results:**

In this reported study, we present evidence that a fraction of the total amount of Rb protein can localize to the mitochondria in proliferative cells taken from both rodent and human cells. This result is also supported by the use of Rb siRNAs, which substantially reduced the amount of mitochondrial Rb, and by acellular assays, in which [^35^S]-Methionine-labeled Rb proteins bind strongly to mitochondria isolated from rat liver. Moreover, endogenous Rb is found in an internal compartment of the mitochondria, within the inner-membrane. This is consistent with the protection of Rb from alkaline treatment, which destroys any interaction of proteins that are weakly bound to mitochondria.

**Conclusion:**

Although a few data regarding an unspecific cytosolic localization of Rb protein have been reported for some tumor cells, our results are the first evidence of a mitochondrial localization of Rb. The mitochondrial localization of Rb is observed in parallel with its classic nuclear location and paves the way for the study of potential as-yet-unknown roles of Rb at this site.

## Background

The retinoblastoma protein (Rb) was the first tumor suppressor protein to be identified [1]. Its loss of function is linked to the development of numerous human cancers [2]. This protein is a major regulator of cell cycle, differentiation and apoptosis. Many of Rb's effects on cell-cycle control derive from its ability to interact with and inhibit the E2F family of transcription factors [3]. Ablation of Rb function in both cultured cells and animals, results, as expected, in deregulated proliferation, but also, more surprisingly, in apoptosis, according to both p53-dependent and p53-independent signaling pathways [4,5]. However, some reports demonstrate that Rb can also act as an inducer of cell death and point to a controversial role for this protein in the regulation of apoptosis [6].

In normal cells, the activity of Rb predominantly depends on the level of phosphorylation of the sixteen potential cdk phosphorylable serine/threonine residues span on the protein [7,8]. It is assumed that the phosphorylation of several critical sites is required to abolish the ability of Rb to interact with E2F factors and to inhibit cell cycle progression. In cancer cells, three main mechanisms account for inactivation of the Rb pathway: genetic inactivation, sequestration by viral oncoproteins (such as T antigen, E1A or E7) or hyperphosphorylation as a consequence of perturbations of cdk activities. Caspase-dependent cleavage may also play a role in Rb regulation in both cancer and normal cells [9-11]. Some recent data suggest that Rb relocation may also regulate its tumor suppressor function, as observed for other tumor suppressor proteins (such as p53), which can be inactivated by a nuclear export mechanism. A nucleocytoplasmic localization of Rb has already been observed for cells with high levels of Rb (MEF-Cdk4^R24C/R24C^) and cytoplasmic sequestration of Rb has been observed in some cancer cells [12,13].

In this paper, we have evaluated the possibility that distinct intra-cellular locations of Rb may account for the contradictory effects of Rb in apoptosis control described in the literature. To this end, we examined the cellular localization of Rb in a range of cell types – tumor or otherwise – of human and rat origin, using several experimental procedures (cell and mitochondria fractionation, cell-free assay), both in the absence and the presence of stress. Surprisingly, we found that a fraction of Rb is localized in the mitochondria of proliferative cells regardless of cell malignancy. This is the first evidence of a mitochondrial localization of Rb. More specifically, Rb was detected in an internal compartment of the mitochondria, within the inner-membrane, data also supported by the incubation of Rb with mitochondria and alkaline treatment.

## Results and discussion

### Mitochondrial Rb is detected by cell fractionation studies

**Figure 1:**
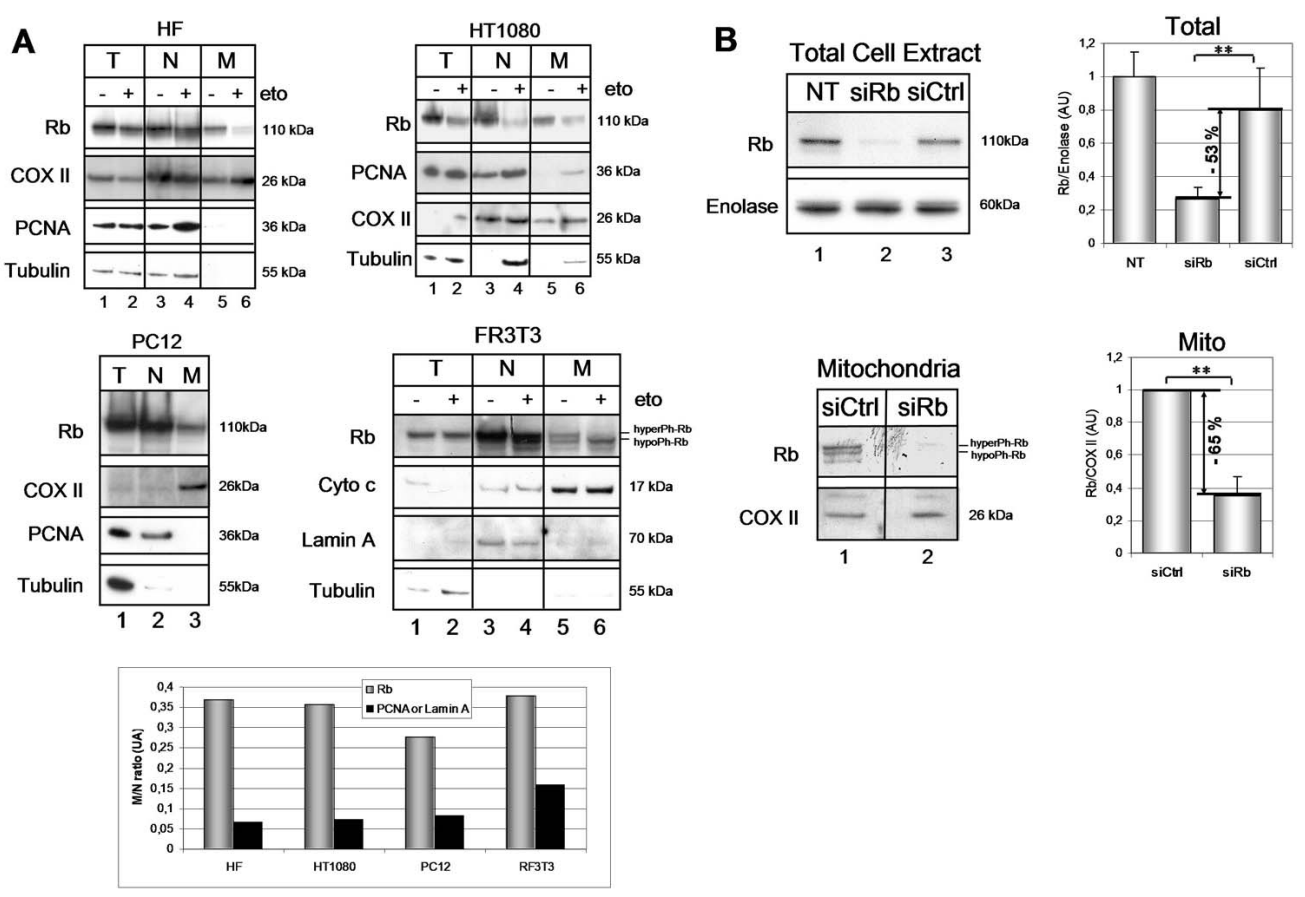
**Rb protein is localized in mitochondria**. **A**. Equal protein amounts (40 μg) of total extract (T), nuclear (N) and mitochondrial (M) fractions of human (HT1080 and HF) and rat (PC12 and FR3T3) cells cultured either untreated or etoposide-treated (16 h), were loaded onto gel and then immunoblotted with: anti-Rb (G3-245) antibody, the mitochondrial (COX II or cytochrome c), the cytosolic (Tubulin) or the nuclear marker antibodies (PCNA or Lamin A). HyperPh or hypoPh represents the phosphorylated state of Rb. (*Lower panel*) The quantification (Image J software) of the Rb and the PCNA protein levels (or Lamin A for FR3T3) in nuclear and mitochondrial fractions illustrated as mitochondrial/nuclear ratio (untreated cells). **B**. Effect of Rb siRNA on the presence of Rb in the mitochondria. FR3T3 cells were incubated for 48 h with Rb siRNA (siRb), control siRNAs (siCtrl) or non-transfected (NT). The total cell extract and mitochondrial fractions (20 μg) were loaded onto gel and subjected to immunoblotting with anti-Rb (G3-245) antibody. Quantifications were performed with respect to Enolase (for the total extract) and COX II (for the mitochondrial extract). Student's tests were performed (**P < 0.01).

Here, we are interested in finding out whether Rb may also be located in other cell compartments, in addition to its conventionally reported nuclear localization. To achieve this, a cellular subfractionation study was first of all conducted in order to isolate enriched mitochondria and nuclei fractions from untreated or etoposide-treated human and rodent cells, such as human primary fibroblasts (HF), human fibrosarcoma cells (HT1080), rat pheochromocytoma cells (PC12) and rat immortalized fibroblasts (FR3T3). Then the total extract (T) and subcellular fractions (N and M) were loaded on gel and analyzed using the Western Blot technique (Fig. 1A). Putative contamination of the mitochondrial fractions was monitored by detecting cytosolic (tubulin) and nuclear (PCNA or lamin A) marker proteins, and antibodies directed against COX II or cytochrome c (mitochondrial markers in living cells) confirmed the enrichment of mitochondrial fractions. Even if nuclear fractions are contaminated to varying degrees by mitochondria, which are often difficult to separate using a specific mitochondria isolation method, mitochondrial fractions are nevertheless not contaminated with either nuclei or cytosolic proteins. In this study, as already outlined, the Rb protein is detected in nuclear fractions of untreated human cells, yet, surprisingly, a fraction of Rb is also detected in the mitochondrial fractions of these cells (Fig. 1A, *lane 3 *and *5*), suggesting that Rb may also be located at this site in parallel with the classically-described nuclear localization. The graph (Fig. 1A, *lower panel*) shows that the ratio mitochondria/nuclei of Rb level in untreated cells is greater than those of PCNA (or Lamin A), indicating that most mitochondrial Rb is not due to a nuclear contamination.

In order to see if this mitochondrial localization of Rb is challenged by apoptosis induction we used etoposide, a DNA-damaging drug acting as a topoisomerase II inhibitor, to activate p53. As described in the literature, the amount of full-length Rb is reduced in nuclear fractions, and this also seems to be the case for mitochondrial Rb (Fig 1A, *lane 4 *and *6*). Nevertheless, the etoposide-treated human or rodent cells display no major change in Rb nuclear or mitochondrial distribution, as observed by the fractionation study.

Next we verified that the protein detected in the mitochondrial fraction was indeed the Rb protein by transfecting FR3T3 cells with Rb siRNAs or with control siRNAs, and then we isolated mitochondria using the same subfractionation method as before (Fig. 1B). Incubation with Rb siRNAs substantially reduced the amount of Rb in the total extract (53%) (Fig. 1B, upper panel *lane 2*), as well as in the mitochondrial fraction (63%) (lower panel *lane 2*). Taken together, these data provide direct evidence that a proportion of total cellular Rb protein is located in the mitochondria in the living cells taken from rodents or humans. Furthermore, as mitochondrial Rb has been detected in primary human cells (HF) (Fig. 1A, upper-left panel *lane 5*), we can suggest that this localization occurs in normal cells and thus is not associated with a transformed character (tumor or immortalized) of the cells we tested.

### Mitochondrial localization of Rb

**Figure 2:**
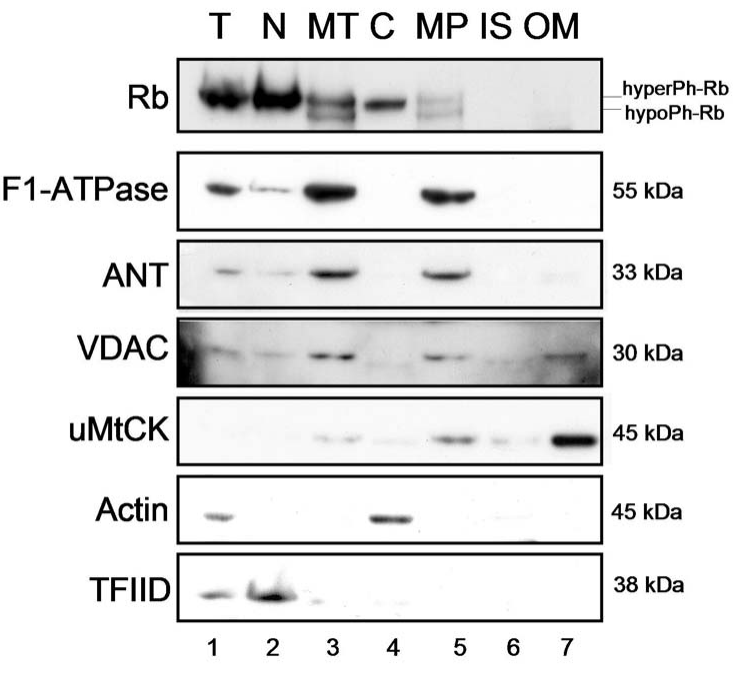
**Rb localization at mitochondrial level**. Rb localization at mitochondrial level: Mitochondria (MT) were isolated from PC12 cells and then subjected to subfractionation into mitoplast (MP) (the inner-membrane and matrix), inter-membrane space (IS) and outer-membrane (OM) using a conventional subfractionation protocol. The total extract (T), the nuclear fraction (N) and the cytosolic fraction (C) were loaded onto gel in parallel. Equal amounts (40 μg) from each fraction were loaded onto gel and submitted to immunoblot analysis using anti-Rb (G3-245) antibody to detect endogenous Rb. Fraction enrichment was tested using antibodies against the β-subunit of F1-ATPase and ANT for mitoplasts, and against VDAC and uMtCK for the outer-membrane. The cytosolic and nuclear contamination was assessed using anti-Actin and anti-TFIID antibodies. HyperPh or hypoPh represents the phosphorylated state of Rb. This data is representative for 2 independent experiments.

Consequently, in order to more accurately determine the exact mitochondrial localization of Rb, we performed a mitochondrial subfractionation study using mitochondria taken from PC12 rodent cells. We used a classic protocol to separate the mitochondrial outer-membrane (OM) from the inter-membrane space (IS) and from the mitoplast (MP, containing both the inner-membrane and matrix compartments) (Fig. 2). All the fractions were then investigated for the presence of endogenous Rb by immunoblot. The enrichments in each mitochondrial subfraction were tested by immunoblotting for β-subunits of F1-ATPase and ANT for the mitoplast fraction and VDAC and uMtCK for the outer-membrane fraction. The contamination of mitochondrial subfractions with cytosolic or nuclear proteins was assessed by detecting Actin and TFIID. As shown in Fig. 2, Rb is once more detected in the total mitochondrial fraction (MT, *lane 3*). At mitochondria, Rb is detected in the mitoplast fraction (MP, *lane 5*) of the mitochondria and is absent in the two other mitochondrial subfractions (IS and OM, *lanes 6 and 7*). These results suggest, firstly, that Rb is located in an internal compartment (either in the inner-membrane or in the matrix) and, secondly, that Rb may be transported across the outer-membrane to its final destination inside the mitochondria. This could be accomplished by an interaction with mitochondrial transporter complexes located on the mitochondrial outer-membrane.

### Study of the *in vitro *interaction of Rb with mitochondria

**Figure 3:**
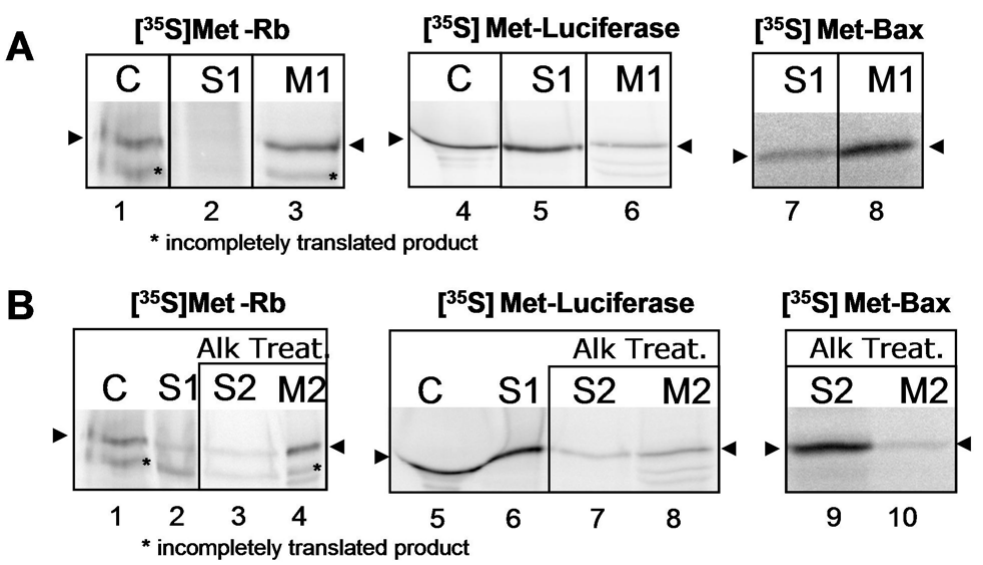
**Rb interaction with mitochondria**. Rb interaction with mitochondria: **A**. [^35^S]Met-Rb, -Luciferase and -Bax proteins produced in vitro were added to purified rat liver mitochondria and then the mitochondria-bound protein fraction (M1) was separated from the non-mitochondria-bound protein fraction (S1), then subjected to gel electrophoresis and autoradiography using a phosphoimager. In vitro translated proteins, were loaded in parallel as a control (C). **B**. In vitro translated [^35^S]Met-Rb, -Luciferase and -Bax were incubated as above and then the mitochondria were alkaline treated (Alk Treat.). *Input*: translated proteins (C); supernatant containing non-mitochondria-bound proteins (S1); supernatant containing detached proteins after alkaline treatment (S2); mitochondria-bound proteins after alkaline treatment (M2). These data are representative for 3 independent experiments.

Afterwards, to further validate these results, we tested the *in vitro *interaction ability of Rb protein with isolated mitochondria in an acellular assay. For this purpose, we prepared vectors expressing the full length of Rb, together with Luciferase (used as negative control) and Bax (used as positive control, in the presence of tBid) (Fig. 3A), and analyzed the binding of the corresponding *in vitro *translated proteins to mitochondria in a cell-free system. The fate of [^35^S]Methionine-labeled proteins was tested by analyzing the interaction with fresh rat liver mitochondria from rodent cells according to the protocol previously described for Bax in the literature [14,15]. As illustrated in Fig. 3A, the Rb protein (*lane 3*, mitochondria-bound proteins M1) is found to bind strongly to mitochondria, similar to Bax binding (*lane 8*); only slight amounts of Rb and Bax remained in the supernatant (*lane 2 *and *7*, non-mitochondria-bound proteins S1). In contrast, the non-mitochondrial protein Luciferase used as negative control barely binds to mitochondria (*lane 6*) and the majority of the Luciferase remains in the supernatant (*lane 5*). This result is consistent with the data from the subcellular fractionation study and suggests that Rb has a high affinity for mitochondrial binding. It is important to note that the interaction of Rb with isolated mitochondria was performed in a buffer, which could enable importation of protein into the mitochondria. Consequently, we cannot exclude the possibility that Rb might be imported into an internal compartment in this study.

To answer this question, but also in order to determine whether Rb is only weakly bound to mitochondria or whether there is a strong interaction, mitochondria pre-incubated with full length Rb produced *in vitro *were subjected to an alkaline treatment. The alkaline treatment is able to destroy any interaction of proteins that are weakly bound to mitochondria. This is the case for the Bax binding previously described [16] and which we used as a positive control for the alkaline treatment (Fig. 3B, *lane 9*). Interestingly, the Rb protein is resistant to alkaline extraction (Fig. 3B, *lane 4*), implying that the interaction between Rb and mitochondria involves strong binding. These data are in agreement with the result of Rb mitoplast localization from the mitochondria subfractionation study, and suggest that Rb may be tightly bound to the mitochondria inner-membrane, either in the inter-membrane space or in the matrix side. Moreover, we cannot exclude the possibility that Rb matrix-side location may play a role in control of the expression of mitochondrial encoded genes, somehow similar to its nuclear activity. The recent discovery of Sankaran et al. concerning the contribution of Rb to the expression of genes encoding proteins of mitochondrial respiration machinery [17], together with our findings concerning the mitochondrial localization of Rb, paves the way for the possibility of Rb involvement in mitochondrial biogenesis and function.

Lastly, our findings concerning the mitochondrial localization of Rb do not exclude its classically observed nuclear localization. Thus, it is important to note that the antibody most widely used in the literature to detect Rb – the G3-245 antibody – recognizes the Rb protein in both nuclear and mitochondrial fractions using the Western Blot method. Conversely, in immunofluorescence studies, the G3-245 antibody we used cannot detect any mitochondrial pattern of Rb (see Additional file 1). This may be explained by the inaccessibility of the antibody to mitochondrial-located Rb, either because of a masked epitope or due to the inability of the antibody to bypass the outer mitochondrial membrane. However, when we quantified the amount of mitochondrial Rb with respect to the total Rb from the Western Blots (Fig. 1A) and then normalized to the same number of cells, we found that mitochondrial Rb level was between 1% to 3% in comparison to the total Rb depending on the cell type. These results may explain why mitochondrial Rb was not previously observed.

## Conclusion

In summary, our results support the presence of a fraction of the total amount of Rb protein in the mitochondria in both rat and human cells. Although some data revealing a cytosolic location of Rb have already been reported for tumors exhibiting a high level of cdk4 activity [12,13], these results are original because, to our knowledge, there is no data in the literature concerning a mitochondrial localization of Rb, with most bibliographic data pointing to a nuclear localization. Nevertheless, this type of location is not exhaustive: we found that most of the Rb was located in nuclear fractions, as previously described. The mitochondrial localization of Rb has been visualized by both cell fractionation and *in vitro *assays. At mitochondrial level, Rb seems to reside inside the organelle inasmuch as it was solely detected in the mitoplast fraction. Altogether, the results present strong evidence for the mitochondrial localization of a small fraction of cellular Rb, in parallel to the nuclear localization classically described, and support the specificity of this interaction.

## Methods

### Cell lines, cell culture and drugs

FR3T3, HF and HT1080 were grown in Dulbecco's modified Eagle's medium (DMEM-F12) supplemented with 100 μg/μl penicillin, 100 U/ml streptomycin, 1% Glutamax and 10% fetal bovine serum under 5% CO_2 _and in a humidified atmosphere. PC12 cells were supplemented with 5% horse serum. For cell death induction, etoposide at a final concentration of 50 μg/ml (Sigma, E1383) was added to freshly plated cultures.

### Western Blot reagents

Western Blot was performed according to the method previously described [18] and the primary antibodies used were: mouse-monoclonal anti-Rb (G3-245, BD Pharmingen), anti-cytochrome c (BD Pharmingen) and anti-F1-ATPase (β-subunit MS503, MitoScience); rabbit-polyclonal anti-Enolase (donated by N. Lamande, College de France, Paris), anti-VDAC and anti-ANT (VDAC and ANT were donated by C. Brenner, UVSQ, Versailles, France); rat monoclonal anti-Tubulin (MAS078, Sera-Lab); goat polyclonal anti-Lamin A (C-20, Santa Cruz), anti-COX II (K-20, Santa Cruz), anti-uMtCK (C-18, Santa Cruz), anti-Actin (sc-8432, Santa Cruz) and anti-TFIID (sc-421, Santa Cruz). The secondary antibodies (peroxidase-conjugated) were anti-mouse, anti-rabbit, anti-rat or anti-goat immunoglobulin (Biosystem). Immunoreactive bands were detected by chemiluminescence using an ECL kit (Amersham).

### Plasmid construction

Wild-type Rb cDNA was subcloned into pGEM-T vectors (Promega) after PCR amplification on a human Rb coding sequence (accession number [EMBL:NM_000321]). The primers used for PCR amplification are 5'-TCTCGAGCGTC**ATGCCGCCCAAAACCCCCC**-3' and 5'-GAAGCTT**TCATTTCTCTTCCTTGTTTGAGGT**-3' (bold: specific Rb sequence). PCR was performed using standard methods with DyNAzyme™ EXT DNA polymerase (Finnzymes), performing 5 PCR cycles at 37°C (denaturation at 94°C for 30 s, annealing at 37°C for 30 s and extension at 72°C for 3 min), followed by 25 PCR cycles at 55°C (denaturation at 94°C for 30 s, annealing at 55°C for 30 s and extension at 72°C for 3 min). The PCR products were extracted from agarose gel using a JETsorb kit and ligated into pGEM-T vector using T4-DNA-ligase according to the manufacturer's instructions (pGEM-T kit, Promega). The PCR products subcloned in pGEM-T were checked by sequencing on both strands using T7 and SP6 primers (MWG biotech).

### Acellular assay of Rb interaction

#### Mitochondria isolation

Mitochondria were prepared from the liver of BD9 female rats, as described earlier [19]. In brief, liver was harvested immediately after animal sacrifice and was homogenised with 5 volumes of an extraction buffer containing 250 mM sucrose, 5 mM Hepes-KOH, pH 7.0, in an Elvehjem motor driven Teflon pestle (20 strokes, 1,000 rpm). Liver homogenate was cleared from cell debris and nuclei by a 1,200 × g centrifugation (20 min) and the crude mitochondrial fraction was pelleted by a 8,700 × g centrifugation (15 min). After two washes by 300 mM mannitol, 10 mM MOPS (pH 7.0) crude mitochondrial fractions were layered onto the top of a discontinuous PercollTM gradient prepared in 300 mM mannitol, 10 mM MOPS (pH 7.0) and consisting of 2 ml of PercollTM 70%, 3 ml PercollTM 30%, 2 ml of PercollTM 18% and 2 ml of PercollTM 10%. Gradients were centrifuged (9,000 × g for 45 min), and different mitochondrial fractions were collected at the different PercollTM interfaces. Mitochondria were washed twice with 300 mM mannitol, 10 mM MOPS (pH 7.0) and used within a maximum delay of 6 h after preparation.

#### In vitro protein synthesis

[^35^S]Met (Amersham Bioscience) labeled proteins were synthesized from cDNA using T7 or SP6 RNA polymerase in vitro transcription followed by translation of the mRNAs in a rabbit reticulocyte lysate (the TNT-coupled transcription/translation system, Promega). The molar concentration of the proteins added to mitochondria was evaluated from the quantity of [^35^S]Met incorporated into the proteins after in vitro translation.

#### Protein incubation with mitochondria

8 fmol of [^35^S]Met-labeled proteins were incubated in the import competent buffer TRB (250 mM sucrose, 80 mM KCl, 10 mM MgCl_2_, 10 mM malic acid, 8 mM succinic acid, 1 mM ATP-Mg^2+^, 20 mM MOPS, pH 7.5), supplemented with 10 mg/ml mitochondria for a 25 μl final volume. The mixture was incubated for 1 hour at 30°C and then centrifuged for 15 min at 8,000 × g at 4°C. [^35^S]Met-Rb, -Luciferase and -Bax binding with isolated mitochondria was analyzed in SDS-PAGE gel and scanned using a phosphoimager (Molecular Dynamics, France). Bax was used in the presence of P13 tBid.

#### Alkaline treatment

Rb, Luciferase and Bax binding with mitochondrial membrane was determined by extraction with alkaline buffer (300 mM sucrose, 0.1 Na_2_CO_3_, pH 11.3) for 30 min at 4°C, followed by centrifugation for 15 min at 8,000 × g at 4°C, analyzed in SDS-PAGE gel and scanned using a phosphoimager.

### Cell fractionation

Mitochondria were prepared using a conventional differential centrifugation procedure as described [20]. Briefly, cells were seeded in 140 mm dishes and incubated at 37°C until they reached 50% confluence, and then were incubated with or without etoposide for the corresponding times. Then, attached and floating cells were collected and washed in TD isotonic buffer (135 mM NaCl, 5 mM KCl, 5 mM Tris-HCl, pH 7.6) and allow to swell for 15 min in ice-cold hypotonic buffer A (250 mM sucrose, 0.1 mM EDTA, 1 mM EGTA, 10 mM Hepes-KOH, pH 7.4, protease inhibitor cocktail AEBSF (Roche)). Cells disruption was performed by passing the cells through a 0.4 × 20 mm needle 10 times. The homogenates were spun at 700 g for 15 min at 4°C and nuclei were separated from the supernatant and resuspended twice in buffer A at a final concentration of 2–3 mg/ml. The supernatant was removed and spun at 7,000 × g for 20 min at 4°C to separate the mitochondrial and cytosolic fractions. The mitochondrial pellets were washed with fractionation buffer B (250 mM sucrose, 5 mM succinate, 5 mM KH2PO4 10 mM Hepes-KOH, pH 7.4) and resuspended in fractionation buffer B to a final protein concentration of 2–3 mg/ml. The protein concentration was determined using Bradford Reagent (Bio-Rad Laboratories). The Rb protein and contamination controls for each fraction were assayed by immunoblot analysis.

### Cell transfections with siRNA

FR3T3 cells were seeded in 140 mm dishes and incubated until they reached 50% confluence. Then, 10 nM Rb siRNA (Rn_Rb1_1_HP siRNA, Qiagen) or negative control (non-silencing, Qiagen) and HiPerFect Transfection Reagent (Qiagen) were mixed in culture medium and then added to the cells, according to the manufacturer's recommendations (HiPerFect Transfection Reagent Handbook, Qiagen). 48 h after transfection, the FR3T3 cells were subjected to cell fractionation (as above) and gene silencing at protein level was analyzed using the Western Blot method.

### Mitochondrial subfractionation

Mitochondrial subfractions were prepared from PC12 cells according to a protocol previously described [21]. First, PC12 cells were seeded in 140 mm dishes and incubated at 37°C until they reached 80% confluence, then cells were collected and washed in a PBS buffer and allowed to swell for 30 min on an ice-cold isotonic buffer 1 (250 mM sucrose, 1 mM EDTA and 10 mM Tris-HCl, pH 7.5, protease inhibitor AEBSF (Roche)). Cells were then Dounce (B piston, 100 times passage) homogenized on ice in buffer 1. Nuclei were pelleted at 1,000 × g and supernatant centrifuged at 6,000 × g for 15 min at 4°C to pellet mitochondria. A portion of mitochondria was kept apart to be loaded on gel in parallel with sub-mitochondria fractions. Mitochondria pellets were resuspended in buffer 2 (0.02 BSA, 20 mM K2HPO4, pH 7.4, protease inhibitor AEBSF) and allowed to swell and Dounce passed (15 times) to disrupt outer-membrane and then mitoplast (sub-mitochondrial particles) were collected after centrifugation at 13,000 × g for 15 min at 4°C. Then the supernatant was ultra-centrifugated for 2 h at 40,000 rpm (50 Ti rotor, OptimaTM LE-80K Ultracentrifuge) at 4°C to separate the outer-membrane in the pellet from the inter-membrane space into supernatant. Mitoplast and outer-membrane fractions were resuspended in Buffer 2. Mitochondria along with the sub-mitochondria fractions were loaded on SDS-PAGE at equal concentrations (40 μg).

## Abbreviations

ANT: adenine-nucleotide translocator; COX II: cytochrome c oxydase II complex; F1-ATPase: F1 portion of ATP synthase; IS: mitochondrial inter-membrane space; MP: mitoplasts; MT: mitochondria total; OM: mitochondrial outer-membrane; PCNA: proliferating cell nuclear antigen; Rb: retinoblastoma protein; uMtCK: ubiquitous mitochondrial creatine kinase; VDAC: voltage-dependent anion channel.

## Authors' contributions

IF carried out the cell and mitochondrial fractionation studies, the siRNA transfection studies, the mitochondria acellular assays and drafted the manuscript. NF participated in the sequence alignment and carried out plasmid construction. MB carried out the immunofluorescence assays. ARE participated in the Western Blot and immunofluorescence assays. VR helped to draft the manuscript. LO and FMV participated in the design of the acellular tests. BM participated in the design of the study. JLV conceived the study, participated in its design and coordination and in drafting of the manuscript. All authors read and approved the final manuscript.

## Supplementary Material

Additional file 1 **Nuclear pattern of Rb in the immunofluorescence study**. The image provided represents the absence of the mitochondrial pattern of Rb protein in the immunofluorescence studies when using the G3-245 antibody. **A**. Untreated human fibrosarcoma HT1080 cells were stained with anti-RbIF8 antibodies (in red) and the nuclei were labeled with Hoechst (in blue). The superimposition of Rb with nuclei is detected in pink in the overlay image. **B**. The same cells were stained with anti-RbG3-245 antibodies (in red); co-stained with mitochondrial marker anti-ANT (in green) and the nuclei were labeled with Hoechst (in blue). No superimposition is detected in the overlay image (no yellow color is visualized). Click here for file
