# Supplementary figures and images for: Evidence for a mitochondrial localization of the retinoblastoma protein
Source: BMC Cell Biol. 2009 Jun 25;10:50. doi: 10.1186/1471-2121-10-50 (PMC2711044; doi:10.1186/1471-2121-10-50)

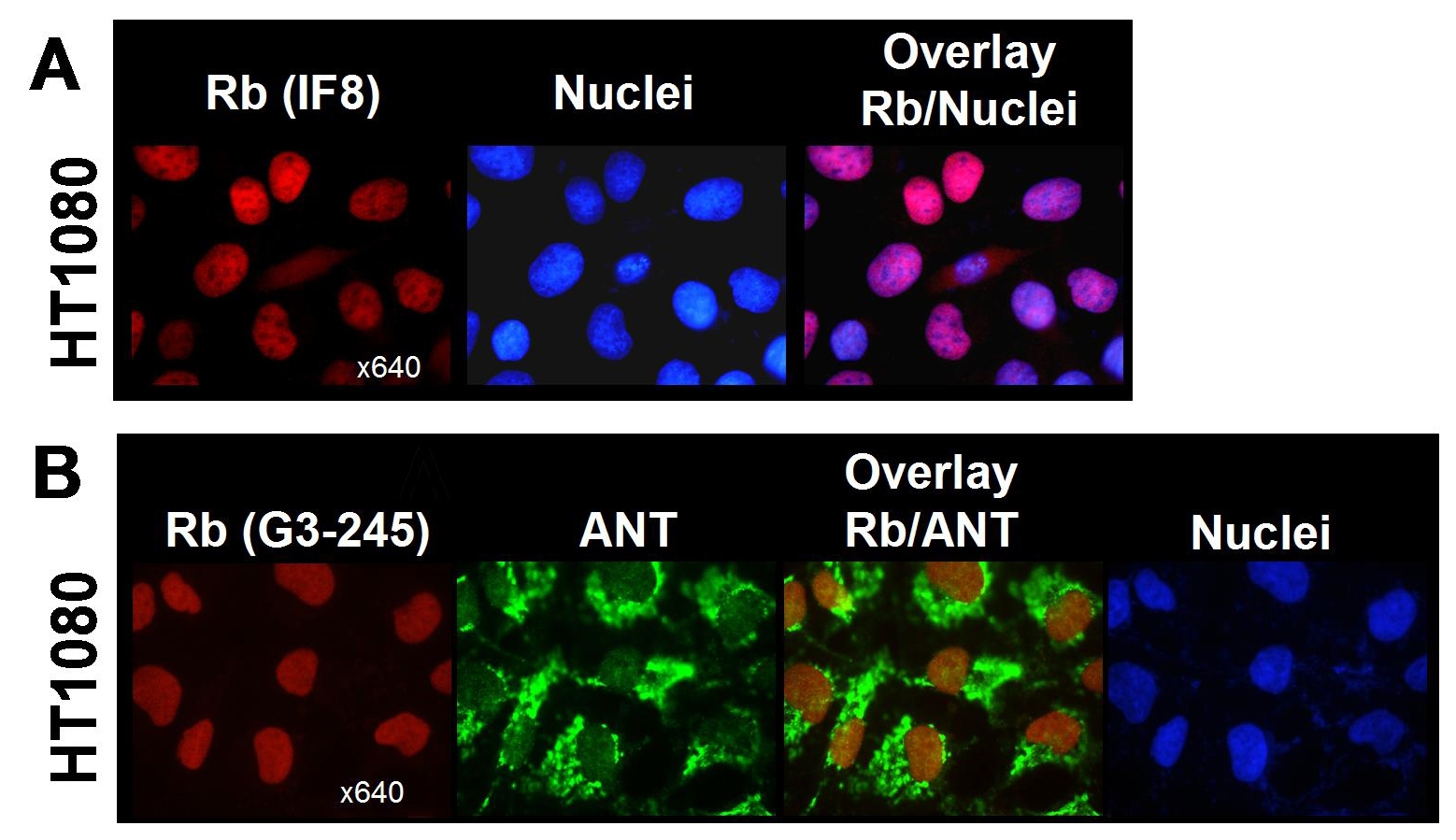

Supplement: Additional file 1 — Nuclear pattern of Rb in the immunofluorescence study. The image provided represents the absence of the mitochondrial pattern of Rb protein in the immunofluorescence studies when using the G3-245 antibody. A. Untreated human fibrosarcoma HT1080 cells were stained with anti-RbIF8 antibodies (in red) and the nuclei were labeled with Hoechst (in blue). The superimposition of Rb with nuclei is detected in pink in the overlay image. B. The same cells were stained with anti-RbG3-245 antibodies (in red); co-stained with mitochondrial marker anti-ANT (in green) and the nuclei were labeled with Hoechst (in blue). No superimposition is detected in the overlay image (no yellow color is visualized). [file 1471-2121-10-50-S1.jpeg]
